# Oral administration of Ketamir-2, a novel ketamine analog, attenuates neuropathic pain in rodent models via selective NMDA antagonism

**DOI:** 10.3389/fphar.2025.1650926

**Published:** 2025-09-02

**Authors:** Itzchak Angel, Rita Perelroizen, Eddy Pichinuk, Erez Aminov

**Affiliations:** ^1^ Mira Pharmaceuticals Inc., Miami, FL, United States; ^2^ Pharmaseed Ltd., Ness Ziona, Israel

**Keywords:** ketamine, Ketamir-2, allodynia, NMDA, neuropathic pain, von Frey, paclitaxel

## Abstract

Ketamir-2 is a novel ketamine analog with improved oral bioavailability and superior safety profile compared to existing ketamine treatments of pain. It is a low-affinity N-methyl-D-aspartic acid (NMDA) receptor antagonist, which selectively binds to the phencyclidine (PCP) site. Ketamir-2 was evaluated in two pharmacological models of neuropathic pain in rats and mice to evaluate its potential in this disease. These tests included the Chung spinal nerve ligation model in rats and mechanical allodynia in a paclitaxel (PTX)-induced neuropathy model in mice. Ketamir-2, administered orally as the pamoate salt, showed significant effects in both tests at variable doses. In these models, it was more effective than orally administered ketamine, pregabalin, or gabapentin, which were used as a positive control.

## Introduction

Ketamir-2 is a new molecular entity and novel ketamine analog, designed with improved oral bioavailability and a superior selectivity and safety profile compared to existing ketamine treatments (Angel et al., 2025). Unlike ketamine, which acts broadly as an N-methyl-D-aspartic acid (NMDA) receptor antagonist, influencing multiple receptor sites, including opioid and monoaminergic systems (Zorumski et al., 2016), Ketamir-2 selectively targets the phencyclidine (PCP) site of the NMDA receptor with lower affinity and does not interact with a large number of other receptors or binding sites (Angel et al., 2025). This selective binding is expected to reduce the risk of adverse effects, such as sedation and dissociation, commonly seen with ketamine (Orhurhu et al., 2023; Hunt et al., 2005), while maximizing therapeutic effects for conditions such as depression, anxiety, and neuropathic pain.

Ketamine and its marketed enantiomer, esketamine, exhibit variable analgesic mechanisms and efficacy in animal models of neuropathic and acute pain. They play significant roles in pain management due to their multifaceted mechanisms, including NMDA antagonism, opioid receptor interaction, and monoaminergic modulation. Ketamine is well established for acute, neuropathic, and chronic pain, particularly in refractory cases, whereas esketamine’s role is emerging, with potential advantages in administration and side effect profile (Mion and Villevieille, 2013; Niesters et al., 2014). Ketamine interacts with opioid receptors, notably the mu-opioid receptor, which likely contributes to its pain-relieving effects (Sarton et al., 2001). Ketamine and its metabolites interact with various neurotransmitter transporters, such as the serotonin transporter (SERT) (Spies et al., 2017) and norepinephrine transporter (NET) (Liebe et al., 2018). By acting as an uptake inhibitor for SERT, ketamine significantly reduces serotonin uptake, with IC_50_ values suggesting a role in its antidepressant effects (Spies et al., 2017). It also inhibits dopamine and norepinephrine uptake, further modulating neurotransmitter activity in the central nervous system (Hess et al., 2021). These multiple binding sites highlight ketamine’s complex pharmacological profile and its potential therapeutic applications beyond anesthesia.

Animal studies confirm the efficacy of ketamine and esketamine in neuropathic pain, primarily via NMDA receptor antagonism (Mei et al., 2010; Christoph et al., 2006; Kawczak et al., 2024), with potentially additional contributions from anti-inflammatory effects (De Kock et al., 2013). Chronic administration yields sustained relief, unlike single doses, and efficacy extends to nociceptive and nociplastic pain, including cancer pain. Esketamine’s higher potency may enhance outcomes, but long-term safety and human translatability concerns remain.

Neuropathic pain, resulting from somatosensory nervous system lesions [e.g., spinal cord injury, complex regional pain syndrome (CRPS), and diabetic neuropathy], is a primary focus of ketamine research. Clinical studies demonstrate that ketamine, particularly via IV infusions, provides rapid but often transient analgesia in neuropathic pain, with strongest evidence for CRPS (moderate evidence for medium- to long-term relief) and weaker evidence for spinal cord injury, fibromyalgia, and mixed neuropathic pain. A 2021 meta-analysis of 18 randomized controlled trials with 706 participants found that IV ketamine (0.15–1.5 mg/kg/day), typically below 10 days, significantly reduced pain intensity in neuropathic pain compared to placebo, but effects were short-lived (up to 1 month) (Pereira et al., 2022). In another study (Bosma et al., 2018) involving 30 neuropathic pain patients, IV ketamine (0.5–2 mg/kg/h, 6 h/day for 5 days) achieved 50% pain relief at 1 month in responders, with pretreatment temporal summation and dynamic connectivity predicting response. This suggests potential for personalized treatment based on sensory testing and brain dynamics.

**FIGURE 1:**
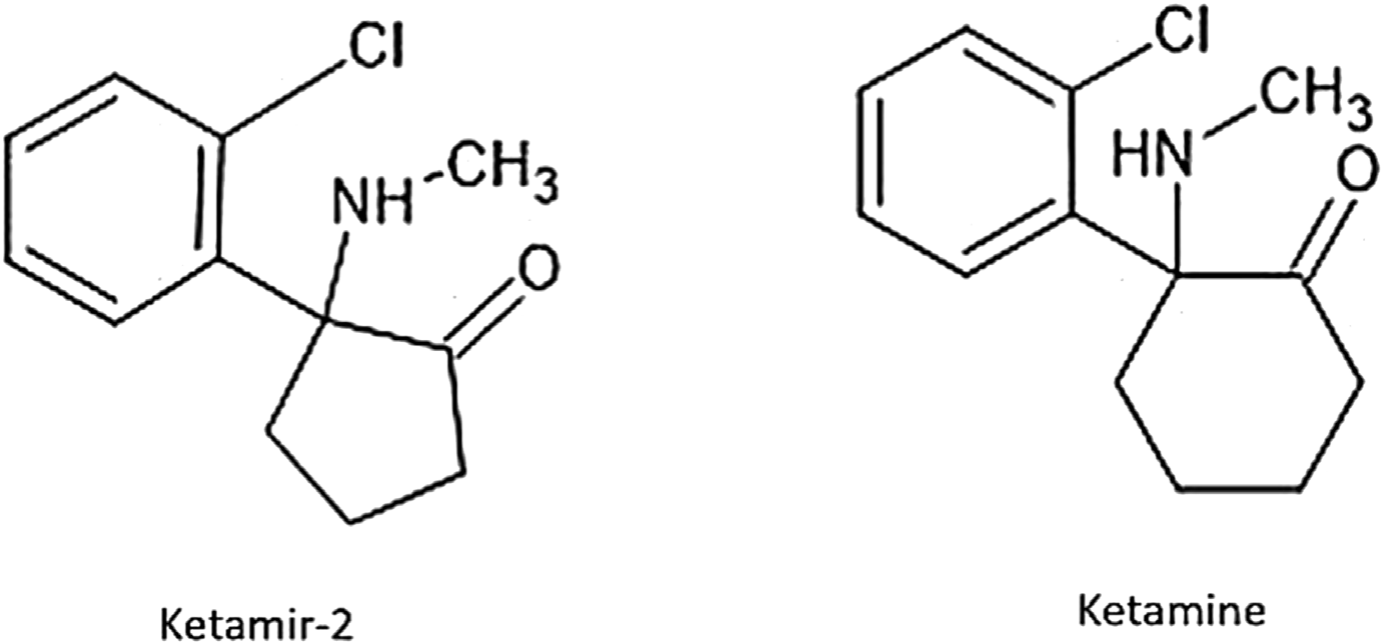
Structures of Ketamir-2 and ketamine.

Ketamir-2 is a five-membered ring analog of ketamine (Figure 1). In these studies, we have used the pamoate salt of Ketamir-2, also known as 1-(2-chlorophenyl)-N-methyl-2-oxocyclopentan-1-aminium 4,4′-hemi-salt of methylenebis (3-hydroxy-2-naphthoate); Ketamir-2; Ketamir hemipamoate. Although Ketamir-2 binds to the PCP site of NMDA, like ketamine, it markedly differs from ketamine not only in its lower affinity to this site but also in its marked selectivity toward numerous other receptor sites and transporters that ketamine also targets. It was found that Ketamir-2 is a low-affinity NMDA receptor antagonist that selectively binds to the PCP site. Its IC_50_ at this receptor site is ∼100 µM. Other sites at the glutamate–NMDA receptor complex were also evaluated. These include the NMDA site, the kainate-binding site, the AMPA site, and the glutamate non-selective ion channel. At all these sites, no significant effects were observed up to 1 mM. In addition, the main metabolite of Ketamir-2, unlike the metabolite of ketamine, retains low PCP-binding affinity and lacks interaction with the NMDA site (Angel et al., 2025).

## Results

### Chung’s model of neuropathic pain in male rats

Ketamir-2, given orally (in all studied as the pamoate salt), was evaluated on two different models of neuropathic pain. In the first model, the Chung’s model of sciatic nerve ligation in male and female rats was used (Chaplan et al., 1994). In this model, the left L5 and L6 spinal nerves were isolated, tightly ligated, and cut with 5–0 silk suture. Subsequently, the rats were evaluated for tactile allodynia using a von Frey Filament (VFF) ranging from the thinnest 0.6 g filament to the thickest 15 g (0.6, 1.4, 2, 4, 6, 8, 10, and 15 g) filament. The von Frey test was performed at baseline (before surgery), day 14 (inclusion/exclusion), day 15, and day 22 (following a single dose of tested item administration). The animals’ body weight was monitored daily after the surgery for a week, and the animals lost some weight as expected after the Chung surgery. Afterward, the animals gained weight. Overall, the groups’ weight did not differ significantly (groups were compared using one-way ANOVA). Male and female rats were tested in different experiments using different comparators. Initially, the study was conducted with male rats, and the female study was subsequently carried out, expanding the comparators. Ketamine was used as a comparator in the male study, whereas pregabalin and gabapentin (100 mg/kg PO each) were used as comparators in the female study.

**FIGURE 2:**
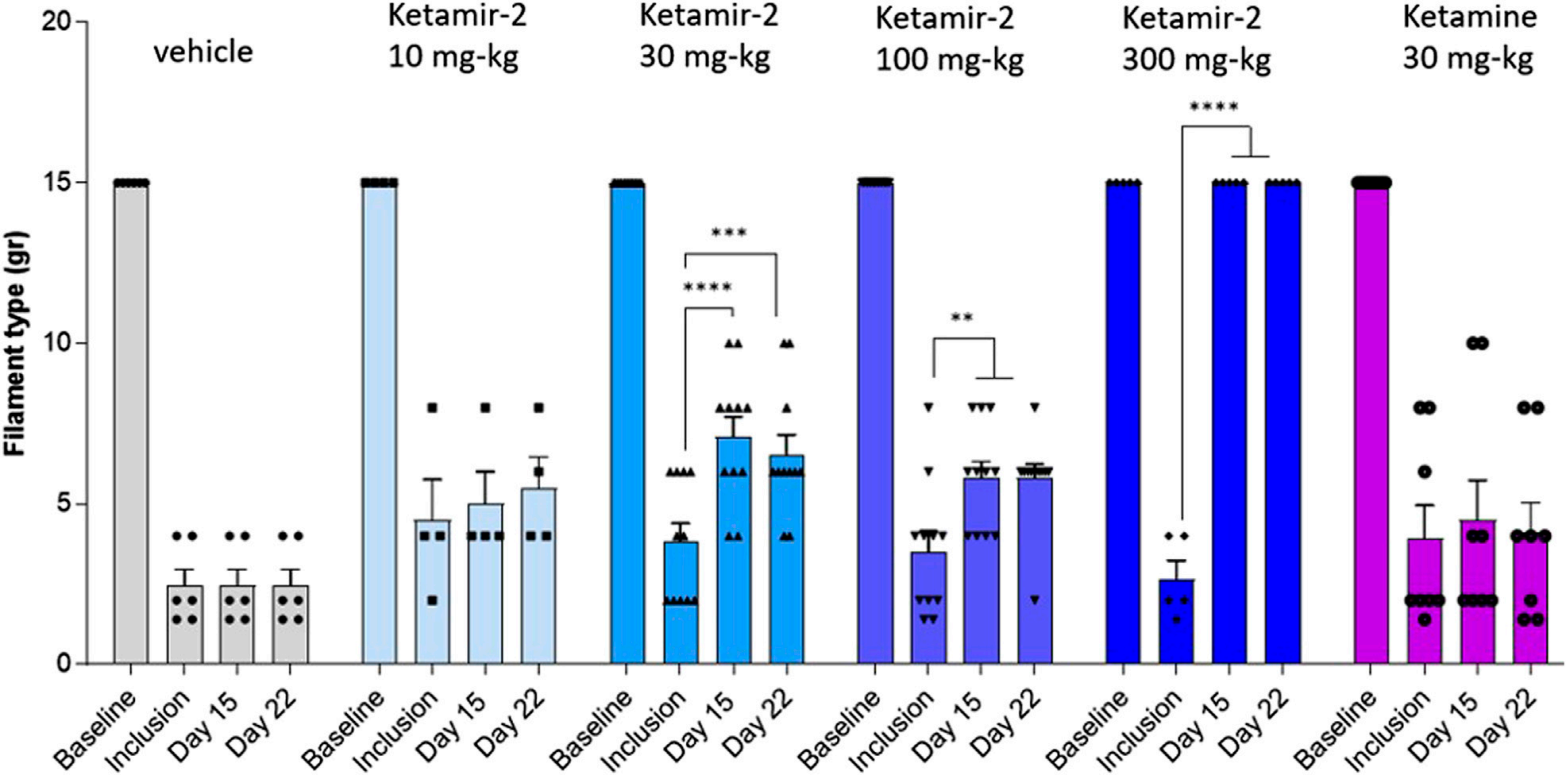
Manual Von Fray test results at days 15 and 22 in male rats (mean ± SEM). The lowest amount of force required to elicit a response was recorded as the withdrawal threshold in grams. The results are presented as means and standard errors for the minimal filament weight response at each group on different time points. Baseline was performed before surgery; inclusion was performed on day 14 following the surgical procedure. VFF test on days 15 and 22 was performed 30–60 min after dosing. Statistical analysis was performed using the paired t-test (see text for significance).

In male rats, a baseline assessment revealed no differences in withdrawal thresholds between groups, with all animals showing no response to the maximum 15 g filament in both hind limbs. On day 14, following test item oral administration, all animals treated with Ketamir-2 at 300 mg/kg (n = 5) exhibited slightly reduced mobility and did not respond to any filament, including the 15 g maximum. As shown in Figure 2, no significant effect was observed at 10 mg/kg (n = 4), but at all higher doses, all groups displayed reduced sensitivity to stimulation following Ketamir-2 treatment. Ketamine, used as a comparator for male rats, did not show any effect at the dose studied (30 mg/kg) (n = 8). Statistical analysis of within-group changes across time points was conducted using paired t-tests. In the vehicle group (n = 6), there was no difference in withdrawal thresholds on days 15 and 22 (after dosing) compared with those at inclusion. In the Ketamir-2 10 mg/kg (n = 4) group, there was no difference in withdrawal thresholds on days 15 and 22 (after dosing) compared with those at inclusion. In the Ketamir-2 30 mg/kg (n = 11) group, the mean withdrawal threshold was significantly higher on days 15 and 22 (after dosing) than that at inclusion: p < 0.0001 and p = 0.0006, respectively. In the Ketamir-2 100 mg/kg (n = 11) group, the mean withdrawal threshold was significantly higher on days 15 and 22 (after dosing) than that at inclusion: p = 0.0072 and p = 0.0037, respectively. In the Ketamir-2 300 mg/kg (n = 5) group, as mentioned above, all animals treated with 300 mg/kg Ketamir-2 showed some reduced mobility after test item administration. The mean withdrawal threshold was significantly higher on days 15 and 22 (after dosing) than that at inclusion, with a p-value < 0.0001. In the Ketamine 30 mg/kg (n = 8) group, there was no difference in withdrawal thresholds on days 15 and 22 (after dosing) compared with those at inclusion. In all groups, the right hind limb, which was not subjected to surgical intervention, was also assessed using the von Frey test. Across all groups and time points, no animals responded to the maximum filament thickness (15 g).

### Female rats

The animals’ body weight was monitored daily after the surgery for a week, and the animals lost some weight as expected after the Chung surgery. Afterward, the animals gained weight. Overall, the groups’ weight in percentage did not differ significantly (groups were compared using one-way ANOVA). Some animals exhibited reduced mobility following dose administration: on days 15 and 22, animals in the pregabalin group and on day 22, animals in the high (300 mg/kg) Ketamir-2 group.

To evaluate mechanical allodynia as a painful sensation stimulated by a light touch, the von Frey test was performed manually at baseline (before surgery), on day 14 (as inclusion criteria), and after dosing on day 15. Both hind limbs were evaluated, with the left limb being the surgical target during the operation. A baseline assessment revealed no differences in withdrawal thresholds between groups, with all animals showing no response to the maximum 15 g filament in both hind limbs.

**FIGURE 3:**
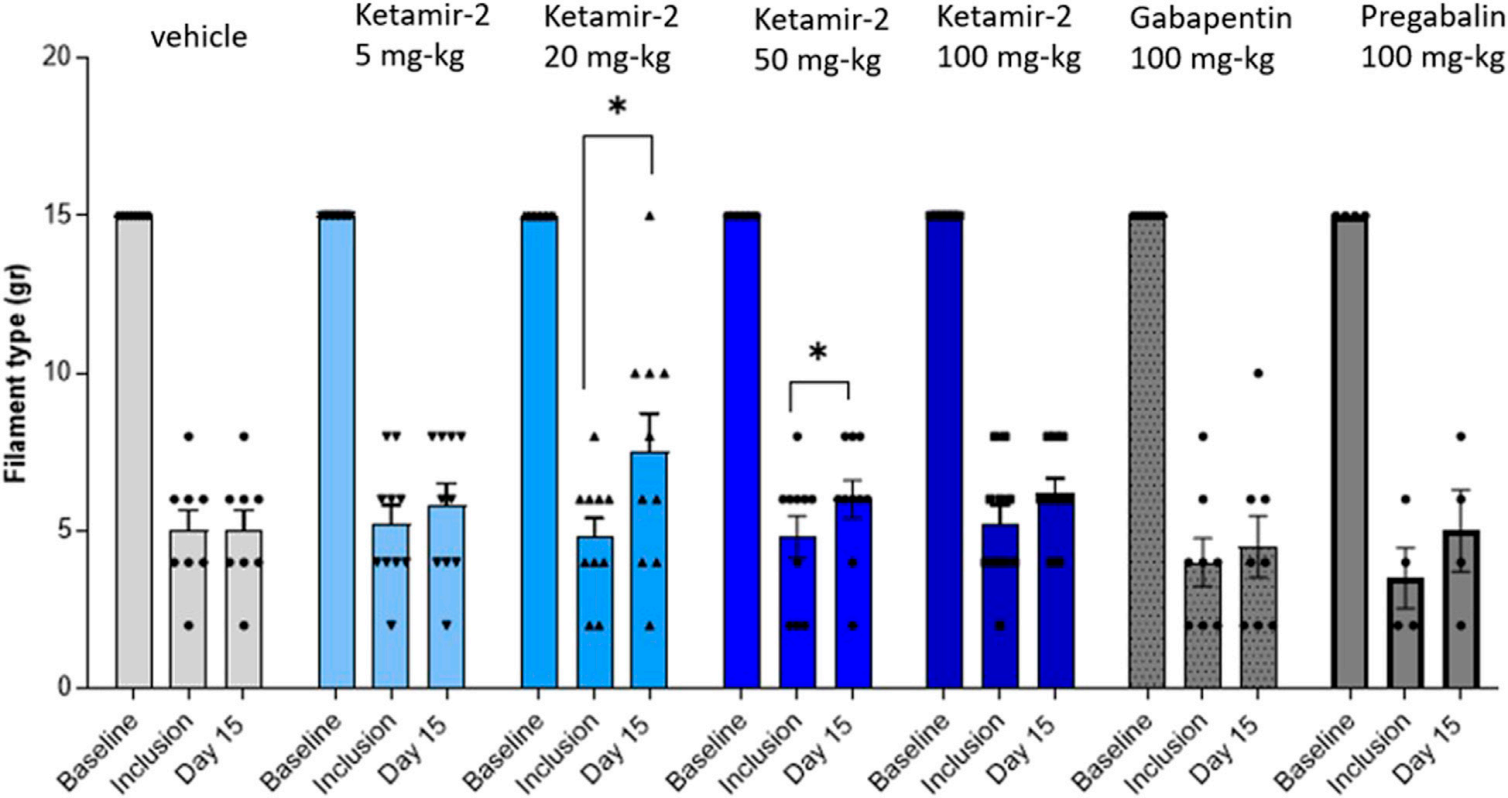
Manual von Fray in female rats, at day 15. Test results (mean ± SEM). The lowest amount of force required to elicit a response was recorded as the withdrawal threshold (in grams). The results are presented as means and standard errors for the minimal filament weight response at each group on different time points. Baseline was performed before surgery; inclusion was performed on day 14 following the surgical procedure and again on day 15. Statistical analysis was performed using the paired t-test (see text for significance).

At day 15, following ligation, statistical analysis of within-group changes across time points was conducted using paired t-tests. In the vehicle group (n = 8), there was no difference in withdrawal thresholds on day 15 (after dosing) compared with those at inclusion. In the Ketamir-2 5 mg/kg (n = 10) group, there was no difference in withdrawal thresholds on day 15 (after dosing) compared with those at inclusion. In the Ketamir-2 20 mg/kg (n = 10) group, the mean withdrawal threshold was significantly higher on day 15 (after dosing) than that at inclusion: p = 0.0257. In the Ketamir-2 50 mg/kg (n = 10) group, the mean withdrawal threshold was significantly higher on day 15 (after dosing) than that at inclusion: p = 0.0239. In the Ketamir-2 100 mg/kg (n = 10) group, there was no difference in withdrawal thresholds on day 15 (after dosing) compared with those at inclusion. In the gabapentin 100 mg/kg (n = 8) group, there was no difference in withdrawal thresholds on day 15 (after dosing) compared to those at inclusion. In the pregabalin 100 mg/kg (n = 4) group, there was no difference in withdrawal thresholds on day 15 (after dosing) compared to those at inclusion (however, it was close: p = 0.0577) (Figure 3). The right hind limb, which was not subjected to surgical intervention, was also assessed using the von Frey test. Across all groups and time points, no animals responded to the maximum filament thickness (15 g).

**FIGURE 4:**
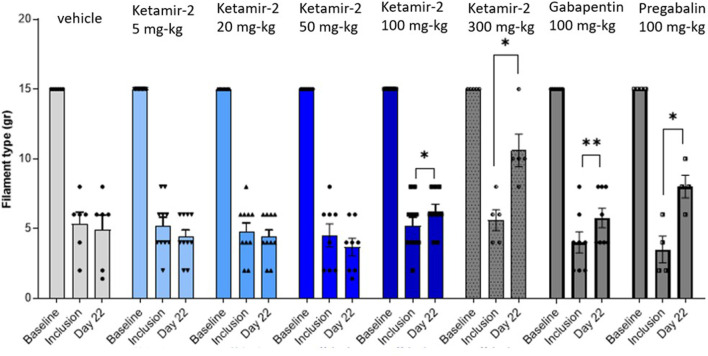
Manual von Fray test results in female rats (Mean ± SEM) on day 22. The lowest amount of force required to elicit a response was recorded as the withdrawal threshold in grams. The results are presented as means and standard errors of the minimal filament weight response for each group on different time points. Baseline was performed before surgery; inclusion was performed on day 14 following the surgical procedure and again on day 22. Statistical analysis was performed using the paired t-test (see text for significance).

Evaluating the results at day 22 in female rats, statistical analysis of within-group changes across time points was conducted using paired t-tests. In the vehicle (n = 6) group, there was no difference in withdrawal thresholds on day 22 (after dosing) compared with those at inclusion. In the Ketamir-2 5 mg/kg (n = 10) group, there was no difference in withdrawal thresholds on day 22 (after dosing) compared with those at inclusion. In the Ketamir-2 20 mg/kg (n = 10) group, there was no difference in withdrawal thresholds on day 22 (after dosing) compared with those at inclusion. In the Ketamir-2 50 mg/kg (n = 8) group, there was no difference in withdrawal thresholds on day 22 (after dosing) compared with those at inclusion. In the Ketamir-2 100 mg/kg (n = 9) group, the mean withdrawal threshold was significantly higher on day 22 (after dosing) than that at inclusion: p = 0.0497. In the Ketamir-2 300 mg/kg (n = 5) group, as mentioned above, animals treated with 300 mg/kg Ketamir-2 showed reduced mobility after test item administration. The mean withdraw threshold was significantly higher on day 22 (after dosing) than that at inclusion, with p = 0.0134. One of the animals exhibited maximal withdrawal latency as before the treatment. This represented the strongest effect observed among all groups. In the gabapentin 100 mg/kg (n = 8) group, the mean withdrawal threshold was significantly higher on day 22 (after dosing) than that at inclusion: p = 0.0062. In the pregabalin 100 mg/kg (n = 4) group, the mean withdrawal threshold was significantly higher on day 22 (after dosing) than that at inclusion: p = 0.0374 (Figure 4). The right hind limb, which was not subjected to surgical intervention, was also assessed using the von Frey test. Across all groups and time points, no animals responded to the maximum filament thickness (15 g).

### Effects of oral administration of Ketamir-2 on neuropathic pain in a PTX-induced pain model in male and female mice

The purpose of this study is to evaluate the effects of Ketamir-2 on neuropathic pain in a PTX-induced pain model using male and female C57BL/6 mice. PTX was injected IP starting on day 1, four times every other day (2 mg/kg). Mechanical allodynia was measured using the von Frey test at baseline (before PTX administration) and on day 8 and 9 as an inclusion/evaluation of pain. On day 9, the von Frey test was performed 30 min after test item administration. There were a total of five oral administrations of test items on day 9 (before VF test) and day 10 (before nesting assessment). The test item was administrated 30 min before each test. Gabapentin was used as a comparator in this study.

#### Male mice

**FIGURE 5:**
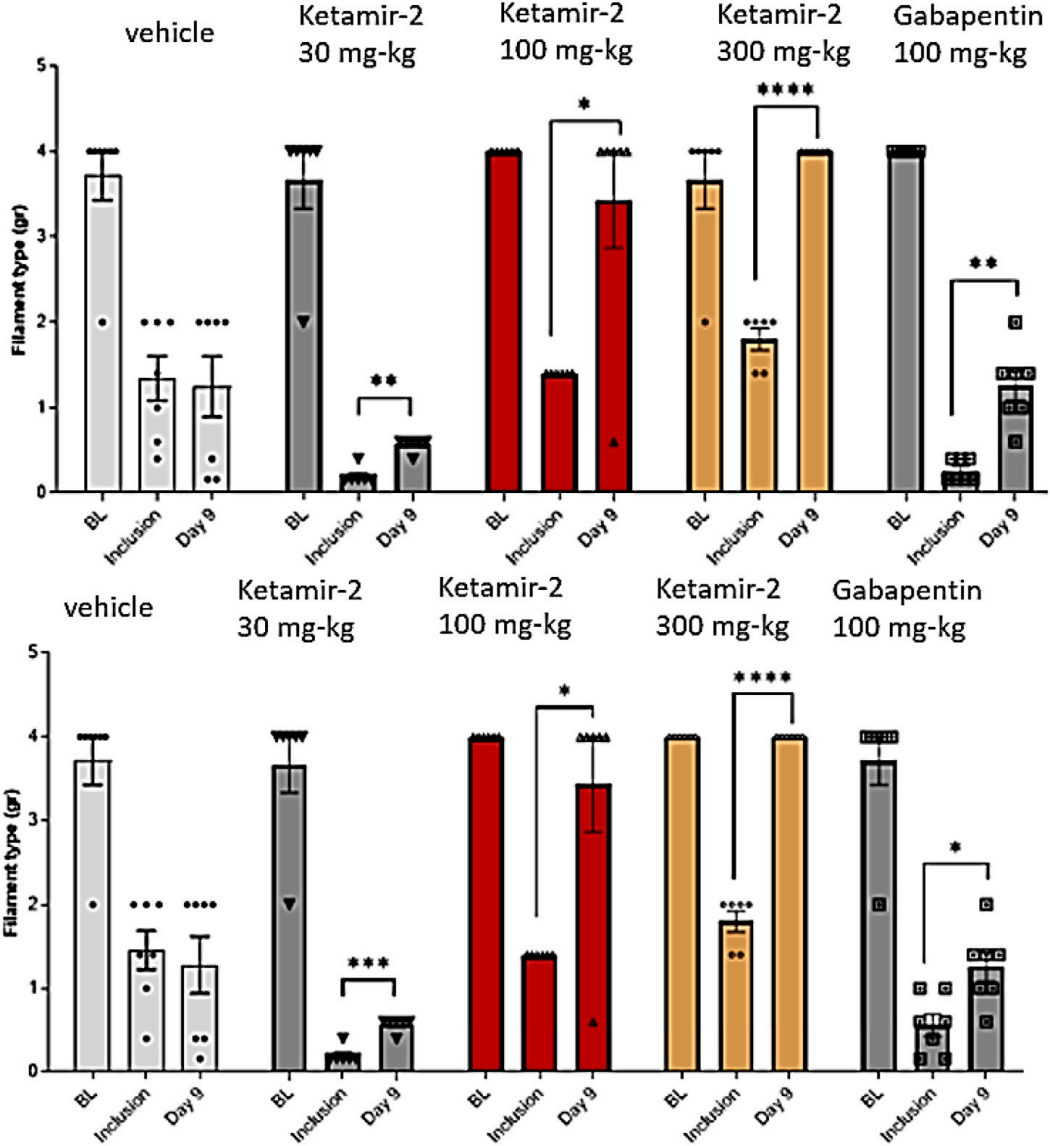
Manual von Fray test results for male mice on day 9 (Mean ± SEM). The lowest amount of force required to elicit a response was recorded as the withdrawal threshold (in grams) and was evaluated in the right and left paws. The results are presented as means and standard errors of the minimal filament weight response for each group on different time points. Baseline was performed before PTX administration; inclusion was performed on day 8 and again on day 9. Data for each paw are presented in top and bottom panels, representing the left and right paws, respectively. Statistical analysis was performed using the paired t-test. *p < 0.02, **p < 0.005, ***p < 0.0005, and ****p < 0.0001.

VFF results compared to the inclusion criteria on day 9 (data are presented in Figure 5) revealed that all groups displayed sensitivity to stimulation following PTX administration on day 8 (inclusion). Statistical analysis of within-group changes across time points was conducted using paired t-tests.

There was no difference in withdrawal thresholds on day 9 (after dosing) compared with those at inclusion in the vehicle group (n = 7). In the Ketamir-2 30 mg/kg (n = 7) group, the mean withdrawal threshold was significantly higher on day 9 (after dosing) than that at inclusion. In the Ketamir-2 100 mg/kg (n = 6) group, the mean withdrawal threshold was significantly higher on day 9 (after dosing) than that at inclusion. In the Ketamir-2 300 mg/kg (n = 6) group, the mean withdrawal threshold was significantly higher on day 9 (after dosing) than that at inclusion. In the gabapentin 100 mg/kg (n = 7) group, the mean withdrawal threshold was significantly higher on day 9 (after dosing) than that at inclusion. The trend of significance was similar for both paws; however, the p-values slightly differed between them.

#### Female mice

**FIGURE 6:**
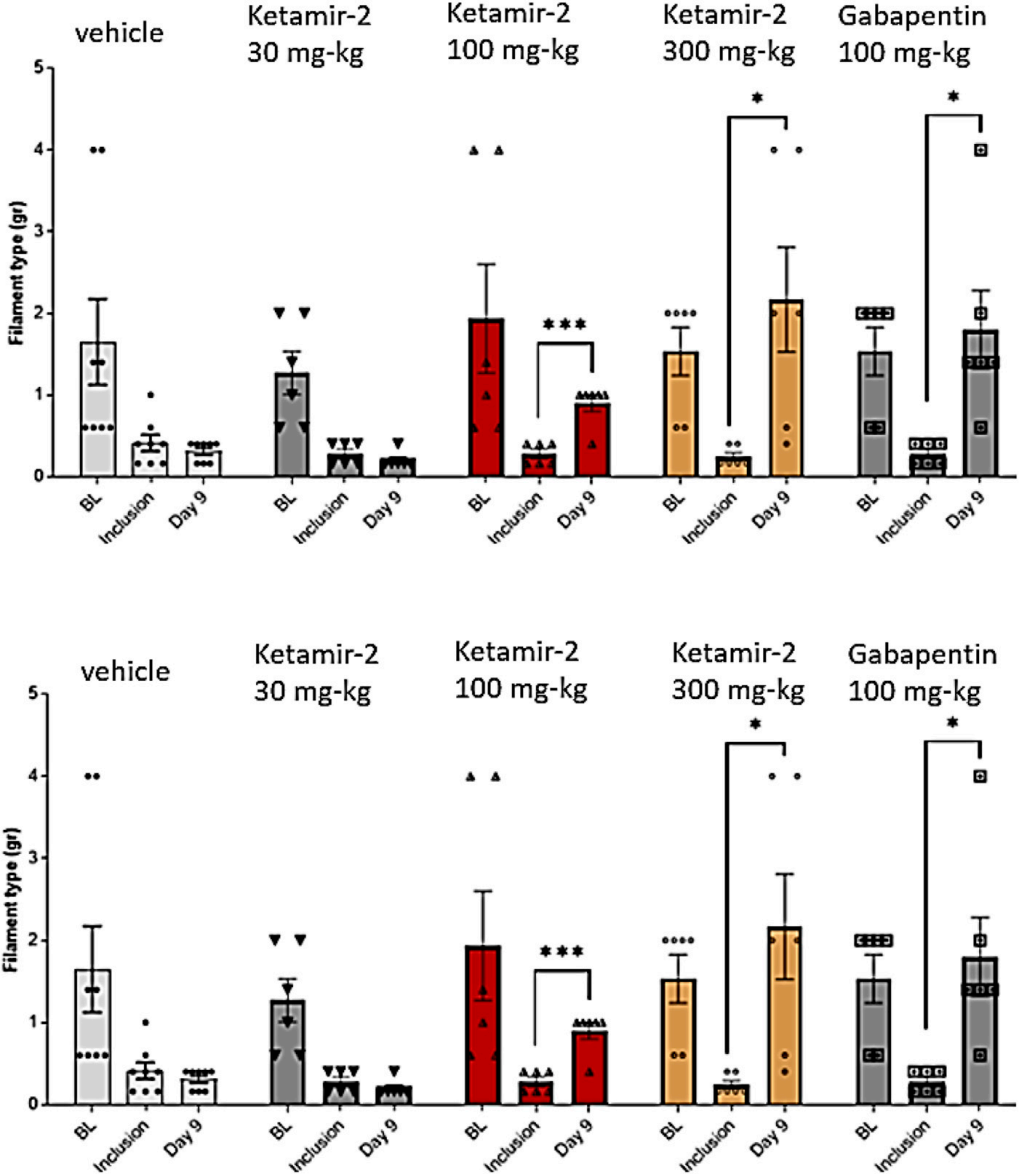
Manual von Fray test results for female mice on day 9 (mean ± SEM). The lowest amount of force required to elicit a response was recorded as the withdrawal threshold (in grams). The results are presented as means and standard errors of the minimal filament weight response for each group at different time points. Baseline was performed before PTX administration; inclusion was performed on day 8 and again on day 9. Data for each paw are presented in top and bottom panels, representing the left and right paws, respectively. Statistical analysis was performed using the paired t-test. *p < 0.05, **p < 0.01, and ***p < 0.001.

VFF results in female mice compared to the inclusion criteria on day 9 (data are presented in Figure 6) revealed that all groups displayed sensitivity to stimulation following PTX administration on day 8 (inclusion). Notably, the baseline response was lower in female mice than in male mice. This indicates a reduced stimulation-evoked response in female mice, suggesting the possibility of lower pain sensitivity in female mice than in male mice. Statistical analysis of within-group changes across time points was conducted using paired t-tests. There was no difference in withdrawal thresholds on day 9 (after dosing) compared with those at inclusion in the vehicle group (n = 8). In the Ketamir-2 30 mg/kg (n = 6) group, the mean withdraw threshold was significantly lower on day 9 (after dosing) than those at inclusion only for the right paw. For the left paw, there was no difference in withdrawal thresholds. In the Ketamir-2 100 mg/kg (n = 6) group, the mean withdrawal threshold was significantly higher on day 9 (after dosing) than that at inclusion. In the Ketamir-2 300 mg/kg (n = 6) group, the mean withdrawal threshold was significantly higher on day 9 (after dosing) than that at inclusion. In the Gabapentin 100 mg/kg (n = 6) group, the mean withdraw threshold was significantly lower on day 9 (after dosing) than those at inclusion only for the left paw. For the right paw, there was no difference in withdrawal thresholds; however, the trend was similar for both paws. The trend of significance was similar for both paws for all groups, except for gabapentin.

## Discussion

These findings are built upon our previously published characterization of Ketamir-2, further supporting its unique therapeutic profile. In two distinct models of neuropathic pain—Chung spinal nerve ligation in rats and paclitaxel-induced allodynia in mice—oral administration of Ketamir-2 produced consistent and robust analgesic effects, outperforming standard-of-care treatments such as pregabalin and gabapentin. Unlike traditional ketamine (Qin et al., 2021), Ketamir-2 exhibited no signs of hyperlocomotion or sedative behavior, reinforcing its favorable safety profile (Angel et al., 2025). These results support continued clinical evaluation of Ketamir-2 as a differentiated, orally available alternative for the treatment of neuropathic pain.

Ketamine is widely studied for its analgesic properties, particularly in acute pain, chronic pain, and neuropathic pain. Its mechanisms involve NMDA receptor blockade, modulation of glutamatergic signaling, and interactions with other systems (e.g., opioid and monoaminergic) (Niesters et al., 2014; Hirota and Lambert 2011; Hirota and Lambert, 2011; Robson et al., 2012; Roth et al., 2013). The comparable pain-relieving effects of ketamine and Ketamir-2, despite Ketamir-2 and its metabolite nor-Ketamir showing low affinity for the NMDA PCP site and no activity at other sites compared to ketamine, indicate that the PCP site plays a critical role in these analgesic effects. In this study, ketamine did not alleviate pain in the Chung model. However, most animal studies report effective pain relief with ketamine when administered parenterally, via systemic or intrathecal routes, due to its poor oral bioavailability (Hayashida and Eisenach, 2010).

Although, in this study, Ketamir-2 has clearly demonstrated dose-dependent and significant efficacy in both ligation and PTX-induced neuropathy models in rats and mice, respectively, both gender- and species-dependent variabilities in the effective doses and amplitude of effects were observed. In male rats, a dose-dependent effect, starting at 30 mg/kg PO, was observed. In this study, a complete reversal of allodynia to basal levels was obtained at the dose of 300 mg/kg PO. No significant effects were observed at the dose of 10 mg/kg PO in this model. Moreover, no differences in the level of pain threshold and effects were observed between days 15 and 22 after the nerve ligation. In rats, some reduced mobility was observed at the highest dose used (300 mg/kg PO). This effect was transient and was not observed in several of the toxicological studies, even at higher doses. Considering that a dose-dependent effect at doses above 50 mg/kg PO was consistently observed in rats, without any motor effects, it seems that the high analgesic effect observed at this dose is genuine and not affected by the reduced motor activity observed at this dose.

In preclinical studies, Ketamir-2 was found to be well absorbed and non-toxic. In rats, the no-observed-adverse-effect level (NOAEL) was found to be 300 mg/kg PO, and in dogs, it was 200 mg/kg PO. The pharmacokinetics of Ketamir-2 was characterized in a series of *in vivo* PK/TK studies conducted in Sprague–Dawley rats and Beagle dogs. PK data indicate that Ketamir-2 crosses the blood–brain barrier with longer exposure times and greater levels of nor-Ketamir present in the brain. Moreover, the PK profile of Ketamir-2 following oral administration is characterized by rapid absorption, a short half-life, and high clearance (data not shown). Brain levels of the drug are expected to affect both its pharmacological activity and its selectivity toward other potential side effects. Studies conducted in rats at high doses have recently confirmed the lack of such side effects, further confirming its good selectivity. However, the female rats had higher systemic exposure than male rats to both Ketamir-2 and nor-Ketamir at 100 and 300 mg/kg/day (data not shown).

Based on this observation, we have initially studied female rats at lower doses in the Chung model. However, no effect was obtained at the low dose of 5 mg/kg PO on day 15, and significant effects were obtained at 20 and 50 mg/kg PO at this time point in female rats. However, these doses were not effective at 22 days post-ligation, and only at the highest doses of 100 and 300 mg/kg PO was a very significant reversal of the effect observed. In parallel, although female rats did not show a significant effect to pregabalin and gabapentin on day 15, they had a significant effect on day 22. We currently do not have a clear explanation for this discrepancy, and we cannot relate it to exposure levels either.

There are documented gender differences in the response to ketamine in rats and mice in pain models, including neuropathic pain models like the Chung model and chemotherapy-induced neuropathy models (e.g., paclitaxel-induced pain). These differences are observed in pain behaviors, analgesic efficacy, and interactions with other drugs, such as opioids. A study by Pascual et al. (2010) investigated gender differences in the Chung model, focusing on ketamine’s analgesic effects in rats with neuropathic pain induced by L5/L6 spinal nerve ligation. They found that ketamine (2–5 mg/kg, intraperitoneal) reduced mechanical allodynia in both male and female rats, but the combination of low-dose ketamine (2 mg/kg) with morphine (2 mg/kg) was more effective in female rats for mechanical allodynia (P < 0.05). Higher doses (5 mg/kg) showed no significant gender differences in mechanical threshold increases. The study suggested that female rats may have enhanced synergistic effects with ketamine–opioid combinations, potentially due to differences in opioid receptor expression or NMDA receptor modulation influenced by estrogen.

In the PTX-model, all groups displayed sensitivity to stimulation, following PTX administration, on day 8 (inclusion). Notably, the baseline response was lower in female mice than in male mice. This indicates a reduced stimulation-evoked response in female mice, suggesting the possibility of lower PTX-induced pain sensitivity in female mice than in male mice. No differences in basal responses were observed between the right and left paws of both genders. Nonetheless, although male mice had a significant reversal of the pain threshold already at the lowest dose studied (30 mg/kg PO), no such effect was observed in female mice. However, at the higher doses of 100 and 300 mg/kg PO, a marked and significant effect was observed in both genders. In addition, the effect seems to be superior to the effect of pregabalin as a complete reversal of sensitivity has been obtained at the highest dose studied with Ketamir-2.

We currently do not have PK data for mice, so an explanation for the differences in sensitivity is lacking. A study conducted by Hwang et al. (2012) on PTX-induced pain in rats has concluded that no gender difference in paclitaxel-induced neuropathic pain or analgesic response to ketamine or morphine was observed in Sprague–Dawley rats. However, low-dose ketamine enhanced the analgesic effect of morphine on paclitaxel-induced mechanical allodynia but only in female rats.

Comparing these results with those obtained previously on antidepressant and antianxiety dose–response activities of Ketamir-2, using the elevated plus maze (EPM), the open-field test, and forced swimming test (FST) in male mice (Angel et al., 2025) have revealed that, at doses above 100 mg/kg, significant effects are also observed in these models.

Taken together, these results suggest that the effective doses for pain relief, along with antidepressant and anxiolytic effects, are in the range of 30–300 mg/kg, with the most consistent results observed in mice and rats at approximately 100 and 300 mg/kg PO.

Although it has been established that NMDA receptors are necessary for the established analgesic effect of ketamine, its additional activities on opioid receptors, notably the mu-opioid receptor, and other sites were postulated to contribute to its pain-relieving effects (Sarton et al., 2001). The current observation that Ketamir-2, which is devoid of these additional activities and is solely a selective PCP site antagonist (Angel et al., 2025), has such potent analgesic properties in two different models and species reinforces the key role of this site in the analgesic activities of these compounds.

Gabapentin and pregabalin are gabapentinoids that bind to the α2δ subunit of voltage-gated calcium channels, modulating neurotransmitter release and neuronal excitability. Their efficacy in animal models of analgesia, particularly for neuropathic and inflammatory pain, has been well documented (Field et al., 1999). In rats, gabapentin (30–100 mg/kg, PO) significantly reduces mechanical allodynia and thermal hyperalgesia in the SNL model (Hunter et al., 1997; Pan et al., 1999). Pregabalin (10–30 mg/kg, PO) shows dose-dependent reductions in allodynia and hyperalgesia in spinal nerve-ligated rats, with greater potency and faster onset than gabapentin. It was, therefore, expected that, at 100 mg/kg PO, a maximal effect would be observed for these reference compounds. Nonetheless, in both rats and mice, Ketamir-2 outperformed both clinically approved and used compounds.

## Materials and methods

### Animals

Animal handling was performed according to the guidelines of the National Institute of Health (NIH), the Association for Assessment and Accreditation of Laboratory Animal Care (AAALAC), and Pharmaseed’s SOPs.

### Chung model

This study was performed at Pharmaseed Ltd., Israel. It was carried out in compliance with “The Israel Animal Welfare Act” and following “The Israel Board for Animal Experiments” Ethics Committee approval # NPC-PL-IL 2404-258.

Sprague–Dawley rats were obtained from Envigo RMS (Israel) Ltd. Male rats had an initial weight of 320–340 g, and female rats weighed 220 g. They were acclimatized for 10 days prior to the study. Animals were housed in polyethylene cages (maximum 3/cage) measuring 42.5 × 26.5 × 18.5 cm, with a stainless-steel top grill facilitating pelleted food and drinking water in a plastic bottle; bedding was prepared using steam-sterilized clean paddy husk (Envigo, Teklad, Laboratory grade, Sani-chips). Bedding material was changed along with the cage at least twice a week. Animals were housed under standard laboratory conditions, air conditioned and filtered (HEPA F6/6) with adequate fresh air supply (minimum 15 air changes/hour). Animals were kept in a climate-controlled environment. The temperature range was 19°C–26°C, and the relative humidity ranged 35%–70%, with a 12 h light and 12 h dark cycle (6 a.m./6 p.m.). The study in male rats was conducted in seven cycles with a total number of 45 rats. The female study was conducted separately in 5 cycles with, overall, 60 female rats. The overall duration of the study was 22 days, after which the animals were euthanized through CO_2_ inhalation.

The surgery was performed under anesthesia with 4% isoflurane in a mixture of 70% N_2_ and 30% O_2_ and maintained with 1.5%–2% isoflurane. Carprofen was given (5 mg/kg) SC before the surgery. As the study monitored pain, no further analgesic treatment was given. A dorsal midline incision was performed at the level of lumbar sacral vertebrae. The left paraspinal muscles were separated from the spinous processes from L4 to S2. The L6 transverse process was carefully removed to visually identify L5–L6 spinal nerves. The left L5 and L6 spinal nerves were isolated, tightly ligated, and cut with 5–0 silk suture. The paraspinal muscles were sutured with 3–0 silk thread, and the skin incision was closed. After acclimation to the room and after test item administration, the rats were placed inside the Plexiglas chamber.

Subsequently, the rats were evaluated for tactile allodynia using a VFF ranging from the thinnest 0.6 g filament to the thickest 15 g (0.6, 1.4, 2, 4, 6, 8, 10, and 15 g) filament. The von Frey test was performed at baseline (before surgery), day 14 (inclusion/exclusion), day 15, and day 22 (following tested item administration). The animals’ body weight was monitored daily after the surgery for a week. To evaluate mechanical allodynia as a painful sensation stimulated by a light touch, the von Frey test was performed manually at baseline (before surgery), on day 14 (as inclusion criteria), and 30–60 min after dosing on days 15 and 22. Both hind limbs were evaluated, with the left limb being the surgical target during the surgery. The smallest filament force that consistently triggers a response (e.g., paw withdrawal in animals) is recorded as the pain threshold. Two to four blinded researchers conducted the measurements in an alternating manner.

A baseline assessment revealed no differences in withdrawal thresholds between groups, with all animals showing no response to the maximum 15 g filament in both hind limbs. All groups displayed sensitivity to stimulation following the surgery.

### PTX model

This study was performed at Pharmaseed Ltd., Israel. It was carried out in compliance with “The Israel Animal Welfare Act” and following “The Israel Board for Animal Experiments” Ethics Committee approval # NPC-Ph-IL-2408–374.

For this study, C57BL mice [obtained from Envigo RMS (Israel) Ltd.] were housed in individual cages separately (up to five per cage) measuring 36.5 × 20.7 × 13 cm with a stainless-steel top grill facilitating pelleted food and drinking water in a plastic bottle; bedding was prepared using steam-sterilized clean paddy husk (Envigo, Teklad, Laboratory grade, Sani-chips). They were acclimatized for 13 days prior to the study. Bedding material was changed along with the cage at least twice a week. All animals were maintained under 12-hour-light/12-hour-dark conditions in groups of up to 10 in polysulfone cages (for the locomotor activity study) and in individually ventilated cages (for OFT, EPM, and FST) with *ad libitum* access to food and water. Every effort was made to reduce the number of mice used and minimize their suffering. A total of 60 mice (30 male and 30 female) were utilized and divided into five groups of 12 animals in each group (6 male and 6 female mice). Male mice had body weights of 24–28 g, and female mice weighed 20–22 g. Overall, the duration of the study was 22 days, after which the animals were euthanized through CO_2_ inhalation.

Paclitaxel was dissolved with Chemophor EL 50%+ ethanol 50% to 6 mg/mL and then diluted to 2 mg/mL in saline. Each mouse was administrated with a final concentration of 2 mg/kg, i. p. Gabapentin was dissolved in PBS. The test item was delivered by the sponsor. Ketamir-2 was resuspended in 5% DMSO, followed by 40% HPβCD (in water). The mixture was subjected to alternating cycles of sonication and vortexing over several hours until a homogeneous suspension was achieved. The stock was then aliquoted to tubes and kept frozen at −20°C until further use. Before each administration, the test item was thawed, sonicated, and diluted to the final concentration per treatment group.

The mice were placed inside the Plexiglas chamber for an acclimation period. Subsequently, the mice were evaluated for tactile allodynia using a VFF ranging from the thinnest filament to the thickest in the following manner: the technician approached the animal from below using the thinnest assorted von Frey filament and touched the hind paw five consecutive times or until the mouse responded. If no response occurred, the next ascending filament was tested. Once a withdrawal response was established, the paw was retested with the preceding descending filament until no response was observed. The lag time between filaments, ascending or descending, was approximately 90 s. Each animal had both hind paws tested in this manner. The lowest amount of force required to elicit a response was recorded as withdrawal threshold in grams. The von Frey test was performed at baseline (before PTX administration), day 8 (inclusion/exclusion), and day 9.

Randomization: allocation to treatment groups was conducted on day 8 according to von Frey test results.

### Statistical analysis

Numerical results were presented as means and standard deviations or standard errors. Whenever applicable, descriptive statistics and group comparisons of data were performed. A probability of 5% (p ≤ 0.05) was regarded as statistically significant.

### Drugs and reagents

The test item was synthetized by Recipharm, Yavne, Israel. In all studies, Ketamir-2 pamoate was used. It was suspended in 5% DMSO, followed by 40% HPβCD (in water). The mixture was subjected to the alternating cycles of sonication and vortexing over several hours until a homogeneous suspension was achieved. The stock was then aliquoted to tubes and kept frozen at −20°C until further use. Before each administration, the test item was thawed, sonicated, and diluted to the final concentration per treatment group. Ketamine was provided by VetVive Vetmarket Ltd. Israel; Gabapentin. Gabapentin was purchased from Tokyo Chemical Industries, Tokyo, Japan; Pregabalin was obtained from Pharmaffiliates Analytics & Synthetics, Panchicula, Haryana, India; Paclitaxel was purchased from Cayman Chemicals. Ann Arbor, Michigan, United States.

## Data Availability

The raw data supporting the conclusions of this article will be made available by the authors, without undue reservation.

## Abbreviations

AI, artificial intelligence; ANOVA, analysis of variance; DMSO, dimethyl sulfoxide; CRPS, complex regional pain syndrome; EPM, elevated plus maze; FST, forced swim test; HP-β-CD, 2-hydroxypropyl-β-cyclodextrin; ND, not determined; NET, norepinephrine transporter; NMDA, N-methyl-D-aspartate; NOAEL, no observed adverse effect level; PCP, phencyclidine; PK, pharmacokinetics; PO, per os (by mouth); PTX, paclitaxel; SEM, standard error of the mean; SERT, serotonin transporter; Std Dev, standard deviation; SOPs, standard operating procedures; TK, toxicokinetics; VFF, von Frey Filament.
